# Determinants of Safety Climate in Industrial Settings: A Systematic Review of Measurement Instruments

**DOI:** 10.3390/healthcare14050596

**Published:** 2026-02-27

**Authors:** Jaqueline Matias da Silva, Antonio Cezar Bornia, Jonhatan Magno Norte da Silva, Rafael da Silva Fernandes

**Affiliations:** 1 Post Graduate Program in Production Engineering, Technological Center, Federal University of Santa Catarina, University Campus Trindade, Florianopolis 88040-900, Brazil; jaqueline.producao@hotmail.com (J.M.d.S.);; 2 Sertão Campus, Federal University of Alagoas, University City, Delmiro Gouveia 57480-000, Brazil; 3 Institute of Integrated Engineering, Campus Itabira, Federal University of Itajubá, Itabira 35903-087, Brazil; rafasfer2@gmail.com

**Keywords:** safety climate, measurement instruments, COSMIN, systematic review, industrial safety, psychometrics

## Abstract

**Background**: Safety climate is widely used to explain and prevent occupational accidents in industrial settings; however, the field remains conceptually fragmented, with multiple measurement instruments coexisting without consensus on the core dimensions that define the construct, limiting the comparability of findings and the effectiveness of organizational interventions. **Objectives**: This study aims to identify, organize, and synthesize the determinants of safety climate reported in validated instruments applied in industrial settings through a systematic literature review. **Methods**: The review was conducted in accordance with the PRISMA 2020 guidelines, with searches performed in the Scopus and Web of Science databases, resulting in the inclusion of 27 empirical studies published between 2015 and 2025. Dimensions reported in the instruments were extracted, grouped by conceptual similarity, and integrated into a common structure. The synthesis examined determinant recurrence across instruments and interpreted the findings in light of the psychometric quality of the measures, as assessed using the COSMIN framework. **Results**: The results indicate that despite the diversity of scales, safety climate determinants derived from measurement instruments consistently converge into four domains: Health and Safety Management, Organizational Safety Resources, Worker Involvement, and Working Conditions. The convergence of these domains across independent instruments, considered alongside the methodological robustness of their validation procedures, indicates a conceptually coherent structural core predominantly supported by instruments with confirmatory structural validation. **Conclusions**: By integrating conceptual structure and measurement quality, this study contributes to reducing fragmentation in the literature and provides an empirical basis for the development, adaptation, and selection of safety climate instruments, with direct implications for research and safety management in industrial environments.

## 1. Introduction

The organizational environment directly influences workers’ safety behaviors, with safety climate among the main predictors of positive occupational health and safety outcomes [1,2]. Safety climate is widely recognized as a key determinant of the prevention of occupational accidents and the promotion of safe behaviors within organizations, particularly in industrial settings characterized by high operational risk. Originally introduced by Zohar [3], the concept of safety climate refers to workers’ shared perceptions of organizational policies, procedures, and practices regarding the value and importance of safety. This construct has been extensively investigated for its ability to explain differences in safety behaviors and safety outcomes across organizational units and industrial sectors [4], and it is also considered a key indicator of the effectiveness of risk management practices and safety management systems in industries across different sectors [3,5].

Empirical evidence indicates that a positive safety climate is associated with reduced accident rates and improved organizational outcomes, whereas a negative safety climate tends to be related to the tolerance of unsafe practices and the prioritization of production over safety [6,7]. In this context, measuring the safety climate is a strategic tool for safety management, provided that it is conducted with psychometrically robust, conceptually coherent instruments [8].

Since its introduction by Zohar [3], safety climate has been operationalized through a wide range of measurement instruments developed across industrial sectors and grounded in diverse theoretical frameworks. Foundational contributions include Griffin and Neal’s [5] model linking safety climate to safety performance outcomes and the Nordic Occupational Safety Climate Questionnaire (NOSACQ-50) [9], which expanded cross-national measurement efforts. Subsequent research has proposed sector-specific instruments for manufacturing, construction, mining, and other high-risk industrial systems, often differing in dimensional structure, item content, and psychometric evaluation strategies. This expanding body of research reflects the conceptual relevance of safety climate as a proactive indicator of accident prevention. However, the diversity of instruments and methodological approaches also raises important questions regarding the rigor, comparability, and robustness of the psychometric evidence supporting these measures.

This methodological and conceptual heterogeneity is particularly critical in industrial contexts, where operational complexity, heavy machinery, multiple shifts, and production pressure amplify occupational risks and the consequences of safety failures. In such environments, conceptually inconsistent or psychometrically weak instruments may generate imprecise diagnoses and compromise the effectiveness of safety interventions. Despite the widespread application of safety climate measures in industrial settings, it remains unclear which dimensions and determinants are consistently supported by robust psychometric evidence.

Previous reviews on safety climate have examined measurement instruments and their relationships with safety outcomes; however, they largely remained descriptive and did not systematically evaluate how determinants are operationalized across instruments within industrial contexts. As a result, conceptual redundancy, overlapping dimensional labels, and inconsistent structural interpretations persist in the field.

Moreover, prior research has rarely integrated conceptual synthesis with systematic psychometric appraisal of the instruments used to operationalize these determinants. The absence of a framework that consolidates determinants while critically examining the methodological robustness of their measurement evidence limits cumulative theory development and hinders comparability across studies and sectors.

Addressing this gap requires a theoretically integrative and methodologically rigorous synthesis focused specifically on industrial contexts.

In light of this need, the present study seeks to consolidate the determinants of safety climate derived from instruments applied in industrial settings and evaluate the methodological robustness of these measures through systematic psychometric appraisal. By organizing determinants within an integrative conceptual structure and interpreting them in light of measurement quality, this study aims to provide a coherent reference for advancing research and improving safety climate assessment in industrial environments.

## 2. Methods

### 2.1. Study Design

This study consisted of a systematic review of safety climate measurement instruments, conducted in accordance with the PRISMA 2020 guidelines [10] and the COSMIN 2018 framework [11]. The review was designed to identify and synthesize the determinants of safety climate reported in instruments applied in industrial settings while systematically appraising the psychometric quality of these measures using COSMIN criteria. The protocol was defined a priori; however, no formal registration was performed, as the review focused on measurement properties rather than intervention effects.

The review was guided by the following research question: “How are safety climate determinants operationalized in measurement instruments applied in industrial environments, and to what extent can they be integrated into a coherent conceptual framework?”.

### 2.2. Eligibility Criteria

Studies were included if they reported the empirical application or validation of a safety climate measurement instrument, explicitly described its dimensions or factor structure, were conducted in industrial production settings, and were written in English.

For the database-driven search, studies published between 2015 and 2025 were considered in order to capture contemporary psychometric practices. However, seminal instruments identified through complementary bibliometric mapping were also included when they met the eligibility criteria and were considered structurally influential for safety climate measurement.

Studies were excluded if they did not measure safety climate as a primary construct, failed to provide sufficient description of the instrument’s dimensional structure, were conducted outside industrial production contexts, or lacked full-text availability.

The restriction to industrial production environments was deliberately adopted to reduce contextual heterogeneity and enhance structural comparability across instruments. Safety climate research spans diverse sectors, including healthcare, transportation, and construction, each characterized by distinct organizational dynamics and risk configurations. The inclusion of highly heterogeneous contexts could obscure determinant-level consolidation and compromise conceptual coherence. Therefore, this review prioritizes contextual stabilization within industrial production systems to support more robust comparative synthesis.

### 2.3. Search Strategy

The literature search was conducted in the Scopus and Web of Science databases, selected for their broad coverage of peer-reviewed journals and relevance to the fields of occupational safety, management, and the social sciences. The search strategy combined descriptors related to the safety climate construct and its measurement using Boolean operators: *“safety climate” AND (“measuring instrument” OR “safety climate questionnaire” OR “safety climate dimensions” OR “validity of the instrument”)*.

The final database search was conducted in July 2025. The complete search strategies used for each database are provided in the Supplementary Materials (Table S1).

### 2.4. Study Selection Process

The study selection process followed the PRISMA 2020 recommendations and was conducted in two stages: (1) screening of titles and abstracts and (2) full-text assessment for eligibility. All stages were performed independently by two reviewers, with disagreements resolved by consensus.

The initial search identified 61 records in Scopus and 131 in Web of Science. After duplicate removal, 166 unique records remained. After full-text assessment, 22 studies met all of the inclusion criteria and were included in the final synthesis.

**Figure 1:**
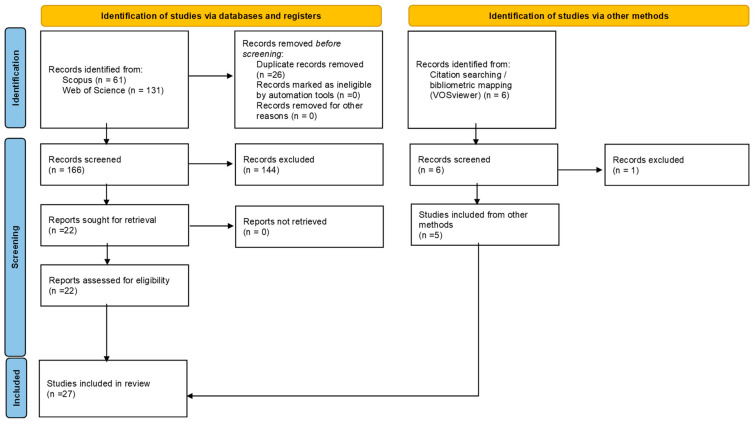
PRISMA 2020 flow diagram of the study selection process.

As a complementary search strategy, bibliometric mapping was conducted using VOSviewer version 1.6.17, to identify influential authors and recurrent publications in the safety climate literature. This procedure identified 6 additional records, of which 5 met the eligibility criteria and were included in the final synthesis, bringing the total to 27 studies. The selection process is illustrated in the PRISMA 2020 flow diagram (Figure 1), which summarizes the identification, screening, eligibility assessment, and inclusion of studies from databases and complementary sources.

### 2.5. Data Extraction

Data extraction was conducted by two reviewers using a predefined standardized protocol. A structured data extraction form was developed a priori to ensure consistency in capturing the study characteristics (authors, year, country, and industrial sector), instrument characteristics (number of items and dimensional structure), psychometric methods employed (e.g., exploratory and confirmatory factor analysis, Item Response Theory), reported measurement properties, and validation evidence.

Both reviewers independently examined each included study and subsequently compared the extracted data to ensure interpretative consistency and alignment with the qualitative synthesis framework. Any discrepancies were resolved through structured discussion until consensus was achieved.

### 2.6. Methodological Quality Assessment Using COSMIN

A formal methodological quality assessment was conducted using the COSMIN 2018 Risk-of-Bias checklist [11], which provides methodological standards for evaluating the quality of studies on measurement properties. The assessment focused on the methodological rigor of the included studies rather than on the intrinsic quality of the instruments themselves.

In accordance with the COSMIN guidelines, the following measurement properties were evaluated where applicable: content validity, structural validity, internal consistency, reliability (test–retest), construct validity (hypothesis testing), responsiveness, and measurement invariance. Not all properties were assessable in every study, as their evaluation depends on specific study designs and statistical procedures (e.g., longitudinal data for responsiveness, multi-group analysis for invariance).

Each measurement property was independently rated by two reviewers using the COSMIN four-level rating system (very good, adequate, doubtful, or inadequate). Ratings were assigned based on compliance with the methodological criteria specified for each domain. In line with the COSMIN “worst score counts” principle, the overall methodological quality rating for each measurement property within a study corresponded to the lowest score obtained on any relevant item within that domain.

Importantly, studies that did not report confirmatory structural validation procedures (e.g., CFA or IRT-based dimensionality assessment) were rated as inadequate for structural validity. Likewise, the absence of explicit content validity procedures (e.g., expert evaluation, cognitive interviewing, or systematic item development) resulted in inadequate ratings for content validity, regardless of the instrument’s theoretical foundation. This approach ensured strict adherence to the COSMIN methodological standards and avoided the overestimation of psychometric robustness.

### 2.7. Qualitative Synthesis of Safety Climate Determinants

A qualitative synthesis based on content analysis was conducted to integrate the factors reported across the different measurement instruments, following three structured stages.

(i) Dimension extraction: All factor, dimension, and subscale names were extracted verbatim from the original instruments.
(ii) Conceptual grouping: Dimensions were grouped according to conceptual similarity, considering semantic equivalence and theoretical function, in order to reduce terminological heterogeneity across instruments.
(iii) Integrative categorization: The resulting groupings were synthesized into broader conceptual categories representing the main determinants of safety climate, while preserving the original theoretical meaning of the factors. The definition of these categories considered both the empirical recurrence of factors and their conceptual relevance, in line with classical and contemporary safety climate models (e.g., [3,5]). For each identified category, the requirements delimiting its conceptual scope were identified and systematized. This process resulted in the definition of four safety climate determinants: Management of Health and Safety, Organizational Safety Resources, Worker Involvement, and Working Conditions.

**Figure 2:**
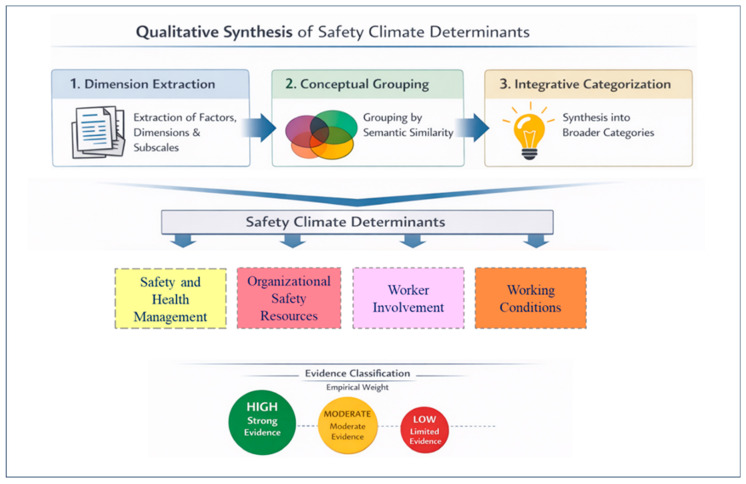
Stages of the qualitative synthesis and integrative categorization of safety climate determinants.

Additionally, the identified safety climate dimensions were analyzed for empirical recurrence across the included studies, allowing for classification of the degree of empirical recurrence (high, moderate, or low) for each determinant. This classification was based on the number of independent instruments reporting each dimension, enabling the distinction between central determinants of safety climate and those with more limited empirical support, and strengthening the interpretation of the qualitative synthesis. The qualitative synthesis process is schematically presented in Figure 2.

Given the conceptual and methodological heterogeneity of the included measurement instruments, no quantitative meta-analysis was conducted. The objective of this review was not to estimate pooled effect sizes or compare intervention outcomes, but rather to identify and integrate safety climate determinant domains derived from instrument structures and supported by psychometric appraisal using COSMIN criteria.

Consequently, no formal assessment of reporting bias (e.g., publication bias) or certainty of evidence (e.g., GRADE) was performed, as these approaches are primarily applicable to reviews synthesizing quantitative effect estimates rather than measurement properties.

## 3. Results

### 3.1. General Characteristics of the Studies

At the end of the selection process described in the previous section, 27 studies comprised the final sample analyzed. The examination of the safety climate instruments employed in these studies constituted the empirical basis for the subsequent identification and synthesis of safety climate determinants. The identified instruments showed substantial variability, with item counts ranging from 7 to 81 and dimension counts ranging from 3 to 11. Application contexts included oil and gas, energy, manufacturing, food processing, and the chemical industry, with a higher concentration of studies conducted in Australia, Iran, the United Kingdom, and China.

**Figure 3:**
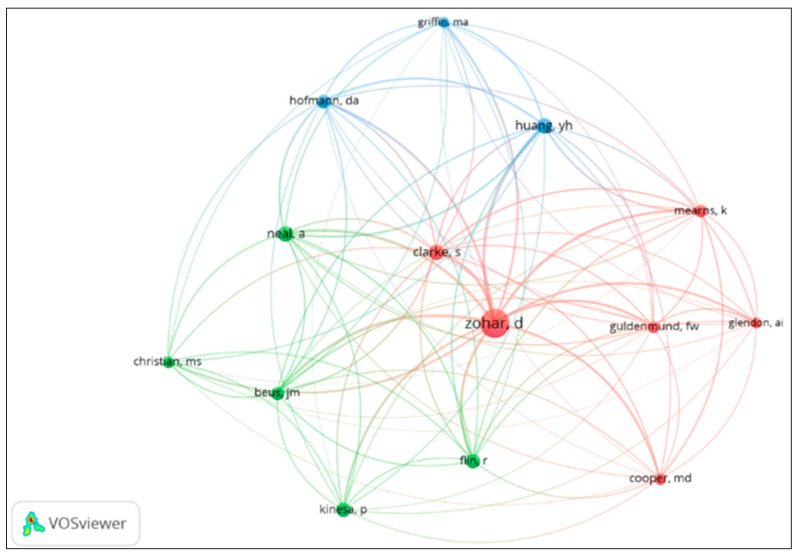
Most cited authors in safety climate studies.

Author citation analysis (Figure 3) highlighted the influence of researchers such as Neal, Griffin, Zohar, and Clarke, who were frequently referenced as the theoretical foundation for safety climate measurement.

It was observed that most of the reviewed studies used instruments such as the NOSACQ-50 [9], Zohar’s original scale [3], and subsequent adaptations [5,12]. These instruments have become international references and continue to be widely applied across different industrial sectors, serving as a basis for cross-study comparisons and for the development of new scales.

Table 1 presents the studies included in the review, highlighting their objectives, number of items and dimensions, application contexts, psychometric methods employed, and overall psychometric quality. This structured characterization provides the analytical foundation for the integrative synthesis of determinants presented in Section 3.3.

### 3.2. Psychometric Properties of the Instruments

The analysis of the psychometric properties of the safety climate instruments included in this review revealed substantial heterogeneity in the validation procedures adopted over time. All studies reported at least one indicator of internal consistency, with Cronbach’s alpha being the most frequently used measure, regardless of the number of items, dimensional structure, or application context. In general, the reported values ranged from acceptable to high. However, in a substantial proportion of studies, these coefficients were interpreted in the absence of confirmed factorial structures, limiting inferences about the internal consistency of the assessed dimensions.

With respect to structural validity, studies focused on the initial development of instruments or the adaptation of existing scales predominantly relied on Exploratory Factor Analysis (EFA) to identify latent dimensions and refine items. In contrast, more established instruments, such as the NOSACQ-50, the Brief NORSCI, and recent short-form versions, reported the use of Confirmatory Factor Analysis (CFA), structural equation modeling, and, in some cases, multilevel analyses, with the systematic reporting of model fit indices. The use of Item Response Theory (IRT)-based models was identified in only one study, in a recent investigation focused on scale reduction and refinement.

Content validity, assessed through expert judgment or structured cross-cultural adaptation processes, was reported in a subset of studies, whereas criterion-related or predictive validity, typically examined through correlations, regressions, or associations with safety outcomes, was reported in only a small proportion of the literature. Although several instruments have been applied across multiple countries, sectors, and occupational groups, formal tests of factorial invariance and cross-group comparability have been reported in only a few recent studies, particularly those employing advanced modeling approaches, and remain absent in most widely used classical instruments.

### 3.3. Integrative Structure of Safety Climate Determinants

Building upon the structured characterization of instruments and their psychometric appraisal presented in Section 3.1 and Section 3.3, the qualitative synthesis integrated the dimensions identified across studies into a coherent set of safety climate determinants.

The extracted dimensions were organized and classified according to their degree of empirical recurrence, determined by the number of independent instruments reporting each determinant. This procedure enabled differentiation between structurally central determinants and those more context-specific or less frequently operationalized.

Table 2 presents the identified determinants, their conceptual requirements, and their respective degree of empirical recurrence. Across the included instruments, safety climate determinants consistently converged into four domains: (i) Management of Health and Safety; (ii) Organizational Safety Resources; (iii) Worker Involvement; and (iv) Working Conditions.

Determinants related to Management of Health and Safety and Organizational Safety Resources exhibited higher levels of empirical recurrence, reflecting their broad representation across independent instruments. The domains of Worker Involvement and Working Conditions also demonstrated consistent empirical presence, although with greater variability in dimensional labeling and operationalization across studies.

The seminal conceptualization proposed by Zohar [3] and subsequently extended by Griffin and Neal [5] emphasized managerial priorities and organizational policy signaling, which informed the domain of Management of Health and Safety. Determinants related to training, competence development, and alignment between organizational practices and safety objectives were grouped under Organizational Safety Resources, representing formal mechanisms through which safety priorities are operationalized within industrial systems.

Theoretical models linking safety climate to safety motivation, knowledge, and performance justify distinguishing Worker Involvement as a separate domain. The included instruments consistently include dimensions related to employee commitment, participation in safety practices, and communication processes, indicating that safety climate reflects not only managerial structures but also workers’ active engagement in safety-related activities.

Dimensions related to operational constraints, production pressure, work pace, and physical working conditions were systematically grouped under Working Conditions. Across the included instruments, these factors captured contextual characteristics of the work environment that shape how safety priorities are experienced in practice. Their recurrence across industrial settings supports recognizing Working Conditions as a complementary structural domain, reflecting the influence of task demands and material conditions on safety-related perceptions.

The methodological appraisal based on the COSMIN Risk-of-Bias checklist (Appendix A) provided complementary information regarding the methodological characteristics of the instruments supporting each determinant. The distribution of empirical recurrence across determinants can therefore be interpreted in light of the psychometric profiles summarized in Appendix A.

**Figure 4:**
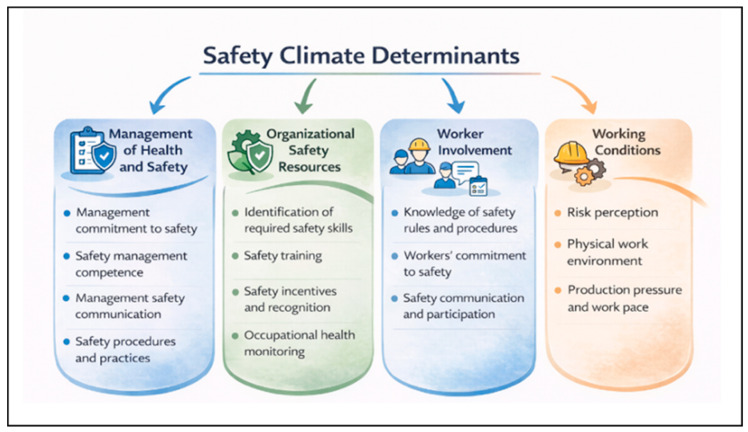
Integrative conceptual structure of safety climate determinants.

The consolidation of factors into these four domains reduced the terminological heterogeneity observed across the analyzed instruments. Figure 4 presents the integrative conceptual structure of safety climate determinants derived from the qualitative synthesis.

## 4. Discussion

### 4.1. Critical Synthesis of Psychometric Evidence

The results of this review reveal substantial heterogeneity in the psychometric procedures used to measure safety climate, reflecting different stages of methodological maturity across the field’s development. Although most studies reported at least one internal-consistency indicator, predominantly Cronbach’s alpha, the systematic evaluation of structural validity, content validity, and cross-group comparability remains limited. This pattern indicates that, in many cases, safety climate measurement has been supported by partial evidence, largely concentrated on internal consistency and exploratory analyses, without the application of more robust confirmatory testing.

A historical analysis of the instruments suggests that part of this heterogeneity stems from the field’s stage of theoretical consolidation at the time these measures were developed. Seminal instruments, such as those proposed by Zohar [3], Griffin and Neal [5], Cooper and Phillips [13], and Zohar and Luria [12], made important theoretical contributions by advancing conceptualizations of the construct and demonstrating its predictive relevance for safety outcomes. However, from a measurement perspective, these instruments were primarily supported by exploratory factor analyses and internal consistency indices, without confirmatory validation of the factorial structure, formal content validity assessment, or invariance testing, thus limiting their psychometric robustness according to current methodological standards established by COSMIN.

In contrast, more recent instruments demonstrate consistent methodological advances, incorporating confirmatory factor analyses, multilevel models, and, in some cases, invariance testing. Studies such as those by Kines et al. [9], Beus et al. [20], Todaro et al. [30], and Summers et al. [31] showed stronger alignment between theoretical grounding, item development, and empirical validation, thereby enhancing the reliability of inferences across groups, organizational levels, and contexts.

The application of more robust modeling approaches, such as those based on Item Response Theory, remains largely unexplored, with such approaches identified in only one study. Similarly, the scarcity of factorial invariance analyses limits comparability across sectors, countries, and occupational groups. Taken together, these findings indicate that, although the field has achieved strong theoretical consolidation, significant methodological gaps persist in the measurement of safety climate, reinforcing the need for evidence-based instruments with greater psychometric rigor and cross-context comparability.

### 4.2. Synthesis of Safety Climate Determinants

The integrative synthesis of safety climate determinants, based on their empirical recurrence across independent instruments, allows for a substantial reduction in the conceptual fragmentation historically observed in the field. Although the literature presents a wide diversity of instruments and terminology, the consolidation of factors into four broad domains indicates the presence of a recurrent structural configuration of the construct observed across industrial sectors and countries of application.

The convergence observed across the domains of Management of Health and Safety, Organizational Safety Resources, Worker Involvement, and Working Conditions suggests that a substantial portion of the variability among instruments may reflect differences in the operationalization of measures rather than explicit theoretical divergence regarding the nature of safety climate. This interpretation indicates that the field has progressed further in diversifying measurement scales than in consolidating a shared conceptual structure, which helps explain the coexistence of apparently distinct dimensional configurations that often capture overlapping content.

Among the identified domains, Management of Health and Safety and Organizational Safety Resources demonstrated higher empirical recurrence, concentrating the determinants most frequently reported across instruments. The predominance of factors related to leadership commitment, managerial competence, and safety communication is consistent with the central role of management in shaping safety climate, in line with classical models proposed by Zohar [3] and Griffin and Neal [5]. These findings indicate that safety climate remains closely associated with workers’ interpretations of organizational priorities as expressed through managerial practices and formal safety management systems.

The domains of Worker Involvement and Working Conditions, although exhibiting greater variability in factor operationalization, represent complementary dimensions for understanding the construct. The recurrence of factors related to participation, voice, and worker commitment highlights that safety climate extends beyond the presence of formal policies and procedures and emerges from daily interactions and opportunities for meaningful engagement in safety practices. Similarly, the incorporation of factors associated with production pressure, work pace, and physical working conditions suggests that more recent instruments increasingly integrate operational aspects that were historically treated as external to the construct, bringing safety climate assessment closer to the realities of industrial work environments.

The coexistence of determinants with different levels of empirical recurrence should not be interpreted as a rigid hierarchy of theoretical importance, but rather as a reflection of the field’s stage of conceptual and methodological development. Determinants with lower recurrence tend to represent emerging, contextual, or sector-specific dimensions whose systematic incorporation into measurement instruments may require further theoretical consolidation and psychometric validation. In this sense, the proposed framework offers a structured reference for guiding future scale refinement, discouraging the proliferation of redundant dimensions and supporting the progressive consolidation of safety climate measurement in industrial contexts.

### 4.3. Conceptual Model

**Figure 5:**
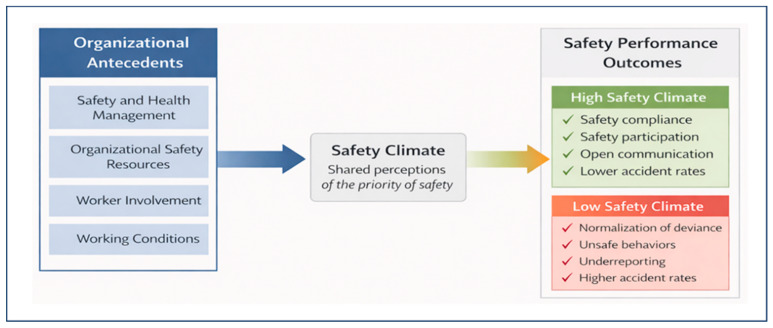
Conceptual model of safety climate determinants.

Based on the conceptual structure derived from the integrative synthesis, this study proposes a model that organizes safety climate determinants across different organizational levels (Figure 5). The model explicates how managerial practices, organizational resources, participatory processes, and working conditions interact in an integrated manner to shape shared perceptions of safety, allowing safety climate to be understood as an interpretive construct that emerges from the interaction between structural and operational elements of the organization.

Factors related to Management of Health and Safety, Organizational Safety Resources, Worker Involvement, and Working Conditions are understood as organizational and operational antecedents that, in an integrated manner, shape workers’ shared perceptions of safety priorities and practices. As illustrated in Figure 5, these determinants link the structural elements of the organization with the conditions and practices of daily work, allowing safety climate to be represented as an emergent construct arising from interactions across different organizational levels.

The proposed model does not establish causal relationships but instead explicates the logic through which managerial practices, formal resources, participatory processes, and operational conditions converge to form the safety climate. This integrative structure enables an understanding of how different organizational domains combine to produce consistent or inconsistent perceptions of the priority attributed to safety, providing a conceptual basis for interpreting variations in safety climate across units, sectors, and industrial contexts.

By organizing determinants into interrelated levels, the model provides an analytical reference for aligning measurement and intervention, supporting both the interpretation of organizational diagnostics and the design of safety management strategies that are more coherent with actual working conditions.

### 4.4. Psychometric Implications for Safety Climate Measurement and Instrument Development

By synthesizing the recurrent determinants of safety climate identified across the included instruments, this study provides an operational conceptual map to support item selection and instrument development. The psychometric quality of the instruments, as appraised using the COSMIN framework, was considered to contextualize the robustness of the measurement evidence underlying each determinant, enabling a more nuanced interpretation of their empirical support.

From a measurement perspective, the integrative framework contributes to a clearer delineation of the construct’s structural core. By consolidating determinants into four conceptually coherent domains, Management of Health and Safety, Organizational Safety Resources, Worker Involvement, and Working Conditions, the framework reduces terminological dispersion and supports greater conceptual alignment across instruments. This clarification strengthens the interpretability and comparability of safety climate assessments conducted in different industrial contexts.

This conceptual delimitation creates favorable conditions for the application of more robust measurement models and for the examination of cross-group comparability, thereby strengthening the methodological consistency and external validity of safety climate assessment. A clearer structural organization also facilitates cumulative research by enabling future studies to build upon a shared conceptual reference rather than generating additional isolated dimensional structures.

Moreover, organizing determinants into broad and conceptually coherent domains enables closer alignment between measurement and intervention. Instruments grounded in this structure can yield more interpretable diagnostics for managers and safety professionals, supporting the identification of priority action areas and the longitudinal monitoring of changes in safety climate.

Finally, by shifting the focus from the multiplication of scales to the consolidation of a common conceptual core, this review contributes to the cumulative advancement of the field, providing an empirical basis for the development, adaptation, and selection of safety climate instruments that reconcile psychometric rigor, theoretical coherence, and practical applicability in industrial environments.

### 4.5. Limitations and Directions for Future Research

This review presents some limitations that should be considered when interpreting its findings. First, the restriction to industrial settings, although intentional to enhance contextual coherence and reduce structural heterogeneity, limits the generalizability of the proposed framework to other sectors such as healthcare, construction, or transportation. Future research could examine whether the integrative structure identified in this review remains stable across non-industrial contexts or whether sector-specific determinants emerge.

Second, the time frame restriction (2015–2025) may have excluded earlier validation studies that contributed to the development of safety climate measurement. This delimitation was adopted to capture contemporary psychometric practices and methodological standards; however, longitudinal analyses comparing earlier and more recent instruments could provide additional insights into the evolution of measurement rigor in the field.

Third, the methodological appraisal relied on the reporting quality of the included studies according to the COSMIN criteria. Because older instruments were developed before the consolidation of contemporary psychometric standards, some seminal studies received lower methodological ratings due to incomplete reporting rather than necessarily weak conceptual foundations. This limitation reflects the evolution of measurement standards over time and underscores the importance of re-evaluating classical instruments using current methodological frameworks.

Despite these limitations, the review offers a structured and transparent synthesis that integrates conceptual and psychometric perspectives, providing a coherent reference for future instrument development and refinement. Further research should prioritize confirmatory and comparative validation studies, as well as cross-sector replication efforts, to strengthen the cumulative development of safety climate measurement.

## 5. Conclusions

This study provides a structured and psychometrically informed synthesis of safety climate determinants in industrial contexts. In response to the persistent conceptual fragmentation observed in the literature (2015–2025), the review consolidates heterogeneous dimensional labels into four coherent domains: Health and Safety Management, Organizational Safety Resources, Worker Involvement, and Working Conditions.

Beyond descriptive aggregation, the present study advances the field by integrating conceptual synthesis with the systematic appraisal of measurement quality using COSMIN criteria. By examining determinant convergence across instruments and interpreting their empirical support in light of methodological robustness, the review distinguishes structurally central domains from more context-specific or less consistently validated dimensions. This integrative approach represents a step toward conceptual stabilization rather than continued scale proliferation.

From a theoretical perspective, the findings indicate that variability across instruments is predominantly operational rather than conceptual, indicating a convergent structural pattern across instruments applied in industrial contexts. From a practical standpoint, the proposed framework offers clearer guidance for instrument selection, adaptation, and development, supporting more precise organizational diagnostics and more strategically targeted occupational safety interventions.

By consolidating determinants within a coherent structural framework and aligning conceptual interpretation with methodological rigor, this study strengthens the evidence base supporting safety climate assessment as a strategic component of occupational health and safety management and provides a cumulative foundation for future research and measurement refinement.

## Figures and Tables

**Table 1:** Empirical studies using psychometric instruments for assessing safety climate.

Reference	Objective	Nº Items/Dimensions	Context/Target Population	Psychometric Methods Used	COSMIN Profile
[3]	To develop and validate a scale to measure the safety climate in industrial settings	40 items/eight dimensions	Metal fabrication, food processing, the chemical industry, and the textile industry	Exploratory Factor Analysis (EFA) to identify the dimensional structure and eliminate items with low factor loadings.	Inadequate
[5]	To investigate the influence of safety climate perceptions on safe work behavior	81 items/10 dimensions	Industrial sector in Australia	Confirmatory Factor Analysis (CFA) using covariance-based structural equation modeling (CB-SEM) to validate the theoretical structure of the instrument.	Adequate
[13]	To examine the relationship between safety climate perceptions and safe behavior, with a focus on training and managerial support	50 items/seven dimensions	Industrial sector in the United Kingdom	Evidence of predictive validity based on correlations and linear regression.	Inadequate
[12]	To develop and test a multilevel model of safety climate	16 items/three dimensions	Metallurgical, food, plastic, and chemical sectors (country not specified)	EFA and internal consistency (Cronbach’s α).	Inadequate
[9]	Researchers developed the Nordic Occupational Safety Climate Questionnaire (NOSACQ-50) to measure workplace safety climate	50 items/seven dimensions	The food industry sector in Sweden and the construction sector in the Nordic countries	EFA, CFA, Rasch, Cronbach’s α, Criterion Validity, and Multilevel Analysis.	Very good
[14]	To develop and validate a scale for the manufacturing industry	45 items/seven dimensions	Workers from Iranian refineries and petrochemical plants	Content validity by experts, CFA, and Cronbach’s α.	Adequate
[15]	To investigate the relationship between workplace safety climate perception, frontline managers’ risk perception, and their involvement in safety management	50 items/eight dimensions	Nuclear power plants in France	Cronbach’s α; no structural analysis reported.	Inadequate
[16]	To investigate the relationships between safety climate, safety behavior, and injuries	21 items/four dimensions	Workers from factories and industrial plants in China	EFA and Cronbach’s α.	Inadequate
[8]	To validate the BriefNORSCI as a brief measure of safety climate	11 items/three dimensions	Workers from the Norwegian offshore oil industry	CFA, RMSEA, CFI, TLI, Construct Validity.	Very good
[17]	To develop and validate the Persian version of the NOSACQ-50 and assess the safety climate among a group of steel industry workers	48 items/six dimensions	Steel industry in Iran	EFA, CFA, Cronbach’s α, and Pearson correlation test.	Inadequate
[1]	To investigate the relationship between safety culture, organizational attitudes, and workers’ well-being.	18 items/eight dimensions	Industrial sector in the United Kingdom	Cronbach’s α; no factor analysis reported.	Inadequate
[2]	To apply IRT to reduce safety climate scales	19 items/four dimensions	Various industrial sectors (country not specified)	IRT with a two-parameter model to estimate item discrimination and difficulty.	Adequate
[18]	To evaluate the effectiveness of the safety climate questionnaire reduction using planned missing data	7 items/four dimensions	Hospital, mining, construction, and oil sectors in Australia	CFA and the multiple imputation technique to reduce items while maintaining predictive validity.	Inadequate
[19]	To assess the safety climate by identifying factors related to safety perceptions and accidents	50 items/seven dimensions	Food industry in Thailand	Cronbach’s α; no structural validation reported.	Inadequate
[20]	To develop and validate a safety climate measure applicable across multiple sectors	30 items/seven dimensions	Various industrial sectors (country not specified)	Construct validity with multilevel CFA and criterion validity via correlations.	Very good
[21]	To assess safety conditions and ergonomic practices in informal footwear workshops	50 items/seven dimensions	Footwear industry in Indonesia	Content validity by experts, Cronbach’s α, and correlations for predictive analysis.	Inadequate
[22]	To quantify the safety climate and analyze the perceptions of workers and supervisors	50 items/seven dimensions	Reforestation industry in Montana, USA	Previously validated instrument (NOSACQ-50); no new psychometric analysis.	Inadequate
[23]	Evaluate the impact of a training program on safety climate	43 items/11 dimensions	Oil and gas industry in Iran	Content validity assessed by experts and Cronbach’s α.	Inadequate
[24]	Analyze the structural factors of safety climate in a chemical plant	43 items/nine dimensions	Chemical industry in Malaysia	Content validity assessed by experts and Cronbach’s α.	Inadequate
[25]	To compare safety climate perceptions across different occupational groups	50 items/10 dimensions	Industrial sector in Poland	Content validity evaluated by experts, EFA.	Inadequate
[26]	To evaluate how leadership, ethics, organizational commitment, and workers’ perceptions influence the implementation of the Zero Accident Vision within the military sector	18 items/10 dimensions	Military industry in Serbia	CFA and Cronbach’s α.	Inadequate
[27]	To validate the NOSACQ-50 adapted to the Thai context and to evaluate how workers and leaders perceive safety issues in the manufacturing and healthcare sectors	42 items/five dimensions	Hospital and business sectors in Thailand	Content validity, Construct Validity through EFA, covariance-based structural equation modeling (CB-SEM), and Cronbach’s α.	Inadequate
[28]	To investigate the safety climate and its correlations with demographic variables	43 items/11 dimensions	Energy sector in the United Kingdom	Cronbach’s α, Spearman correlation, and Descriptive statistics.	Inadequate
[29]	To investigate employees’ perceptions of the safety climate through a mixed-methods approach	11 items/11 dimensions	Workers from the medical equipment sector in Malaysia	CFA and Qualitative Content Analysis.	Inadequate
[30]	To develop a safety climate scale for organizations with high management maturity	40 items/eight dimensions	Energy, oil, and manufacturing and printing sectors in Italy	Construct validity via EFA and CFA; convergent and discriminant validity based on the Fornell–Larcker criterion.	Very good
[31]	To validate a shortened version of the NOSACQ-50 for continuous monitoring of safety climate	24 items/seven dimensions	Various industrial sectors in Australia	CFA; Validity through Pearson correlations.	Inadequate
[32]	To investigate critical factors such as training and accident reporting in critical events	49 items/three dimensions	Cement industry in Nigeria	EFA with PCA and Varimax rotation and Cronbach’s α.	Inadequate

Note: The COSMIN rating presented in this column refers to the methodological adequacy of the procedures used to validate the measurement properties. The classifications “Very good”, “Adequate”, and “Inadequate” indicate different levels of compliance with COSMIN methodological standards and do not represent an evaluation of the overall scientific quality or theoretical relevance of the studies, but exclusively the rigor of the reported measurement procedures.

**Table 2:** Mapping of safety climate determinants and conceptual requirements to supporting studies.

Category of Determinants	Conceptual Requirement	Empirical Recurrence	Studies That Reported the Determinants
Health and Safety Management	Management commitment to safety	High	[1,3,4,5,8,13,14,16,17,20,24,28,29,30,31,32,33]
	Safety management competence	High	[4,5,8,14,17,31,33]
	Management safety communication	High	[1,2,3,5,20,30,31]
	Safety procedures and practices	High	[1,14,18,28,30,33,34]
	Identification of required skills	Low	[5,14,20,31]
Organizational safety resources	Safety training	High	[1,2,5,13,14,16,18,20,24,28,29,30,32,33,34,35]
	Safety incentives and recognition	Low	[3,5,20,28,30]
	Occupational health monitoring	Low	[1,13,25,29]
Worker involvement	Knowledge of safety rules and procedures	Low	[5,14,20]
	Workers’ commitment to safety	Moderate	[3,5,17,20,24,29,31]
	Safety communication and participation	Moderate	[1,2,3,5,20,30,32]
Working conditions	Risk perception and acceptance	Moderate	[1,13,15,17,27,29]
	Physical work environment	Moderate	[1,3,24,25]
	Production pressure and work pace	Moderate	[3,13,25,28,29]

## Data Availability

No new data were created or analyzed in this study.

## Supplementary Materials

The following supporting information can be downloaded at: https://www.mdpi.com/article/10.3390/healthcare14050596/s1. Table S1: PRISMA 2020 Checklist.

## Appendix A

This appendix presents the detailed methodological appraisal of the psychometric properties of safety climate instruments using the COSMIN 2018 framework. The evaluation focuses exclusively on the adequacy of the measurement procedures reported in each study, considering content validity, structural validity, internal consistency, reliability, construct validity, responsiveness, and comparability. Classifications reflect compliance with COSMIN methodological standards and do not represent an assessment of the theoretical relevance, empirical contribution, or practical importance of the instruments. Studies were rated as inadequate when key measurement properties (e.g., content validity or confirmatory structural validation) were not formally evaluated, in accordance with the COSMIN guidelines.

**Table A1:** Methodological Quality Appraisal of Safety Climate Instruments Based on COSMIN Criteria.

Reference	Content Validity	Structural Validity	Internal Consistency	Reliability (Test–Retest)	Criterion/Construct Validity	Responsiveness	Invariance/Comparability	Overall Quality
[3]	Inadequate No formal content validation; items derived from theoretical frameworks only.	Inadequate Early exploratory evidence only; no CFA/IRT/invariance testing.	Inadequate Internal consistency not reported.	NA	Adequate Associations with safety inspections are consistent with the theory.	NA	NA	Inadequate Strong theoretical contribution, but structural, content, and reliability analyses are limited.
[5]	Inadequate Solid theoretical model and systematic conceptual development, but with no formal testing by experts and/or the target population.	Adequate Hierarchical CFA supported; no invariance or IRT analyses.	Adequate α ≥ 0.70 across dimensions with CFA-confirmed structure.	NA	Very good Validated relationships with safety performance, motivation, and knowledge.	NA	NA	Adequate Robust CFA-based structure and construct validity; limitations in content validity and invariance.
[13]	Inadequate Items based on literature and previous experience, but without systematic expert or target-population validation.	Inadequate EFA conducted; structure not confirmed by CFA.	Inadequate α around 0.70–0.80 across dimensions; no confirmatory testing of structure.	NA	Adequate Predicts safe behavior; limited hypothesis testing.	NA	NA	Inadequate Solid theoretical basis, but lacks item development, content validity testing, and CFA-based structural validity.
[12]	Inadequate Strong theoretical basis, but no structured expert review or cognitive testing.	Inadequate Strong EFA with a large sample; no CFA.	Inadequate Internal consistency acceptable; no CFA.	NA	Adequate Validation of multilevel effects on safety performance.	NA	NA	Inadequate Theoretically grounded multilevel model, but psychometrically inadequate.
[9]	Very good Strong theoretical foundation and iterative expert-based development, complemented by face validity testing with lay participants.	Very good EFA, CFA, and Rasch analysis applied.	Very good α > 0.80 across all dimensions, and Rasch analysis supports internal consistency.	NA	Very good Predicts motivation, safety perceptions, and behaviors.	NA	NA	Very good Strong psychometric evidence and Rasch analysis; however, no full invariance or test–retest reliability.
[14]	Adequate Expert evaluation conducted; I-CVI > 0.78 indicates systematic content validation.	Adequate PCA and CFA with acceptable fit (RMSEA 0.05–0.06; CFI 0.85); PCA instead of FA and CFI < 0.90.	Very good global α = 0.96; subscales 0.62–0.93; interpreted after CFA.	Adequate ICC 0.93, but small sample *(n* = 26).	Adequate external comparisons of accident history.	NA	NA	Adequate Strong structure and reliability, but inadequate construct validity determine the overall rating.
[15]	Inadequate No item development, no expert review, no cognitive testing, or insufficient reporting.	Inadequate Structure confirmed, but without robust EFA/CFA.	Inadequate α between 0.70–0.80; structure not confirmed by CFA.	NA	NA	NA	NA	Inadequate Theoretically interesting, but does not meet COSMIN standards for measurement property evaluation.
[16]	Inadequate The study derived items from classical safety climate dimensions but did not report expert panel or target population evaluations.	Inadequate The study described a clear factorial structure but omitted CFA and invariance testing.	Inadequate α > 0.85; robust internal consistency; no CFA.	NA	NA	NA	NA	Inadequate No item development, no content validity, only EFA (no CFA), internal consistency not interpretable, and no construct validity testing.
[8]	Very good. Developed from an extended version validated by experts.	Very good Strong CFA with a large sample and good fit indices.	Very good α > 0.85; strong convergent validity.	NA	Very good Associations with sleep, health, and shift work.	NA	NA	Very good Strong structural validity, high internal consistency, and robust theoretical and empirical support.
[17]	Inadequate Only translation/face review; no COSMIN content-validity assessment.	Very good Solid factorial structure, excellent fit indices, adequate sample size.	Very good Global α = 0.94; strong consistency within dimensions.	NA	NA	NA	Adequate Comparability with the original version.	Inadequate Good CFA and reliability, but lacks content validity, invariance, test–retest, and construct hypotheses.
[1]	Inadequate Used pre-existing instruments without evaluating item relevance, so there is no content validity.	Inadequate Structure based on predefined cultural dimensions; no CFA.	Inadequate Acceptable internal consistency across nine dimensions; no CFA.	NA	NA	NA	NA	Inadequate The article is not a psychometric study and does not evaluate any measurement properties of the instruments used.
[2]	Adequate Items adapted from Zohar & Luria and refined with IRT, supported by expert-based selection.	Very good IRT (GRM) is used to select items with high information and strong structural evidence.	Very good α ≥ 0.89; IRT-based item refinement supports internal consistency.	NA	Very good High correlations with the full-scale version.	NA	Very good IRT supports cross-version comparability.	Adequate Strong and innovative psychometric evidence.
[18]	Inadequate The studies did not document new expert evaluations or content assessments, as they relied solely on previously validated instruments.	Very good EFA and CFA confirmed a multidimensional structure.	Very good α > 0.80 across dimensions; CFA supports factor structure.	NA	NA	NA	NA	Inadequate Inadequate due to lack of content validity, measurement error, reliability, and invariance.
[19]	Inadequate Translation/back-translation performed, but no formal expert analysis of relevance/comprehensiveness.	Inadequate Structure supported by EFA, but no CFA.	Inadequate α between 0.75 and 0.84; no CFA confirmation.	NA	NA	NA	NA	Inadequate No content or structural validity; internal consistency not interpretable.
[20]	Very good Strong theoretical foundation; extensive expert review and systematic development.	Very good CFA confirmed a robust second-order model with excellent fit.	Very good α between 0.94 and 0.98; CFA confirms structure.	NA	Very good A meta-analysis confirmed that safety climate is a robust predictor of accidents, injuries, and safety behaviors.	NA	NA	Very good Excellent structural, internal, and construct validity; strong invariance evidence across samples.
[21]	Inadequate Combined use of validated instruments; no new content validation.	Inadequate The study applied existing scales without conducting a structural analysis.	Inadequate α between 0.75 and 0.84; no CFA confirmation.	NA	NA	NA	Inadequate Only mean comparisons; no MG-CFA, DIF, or IRT.	Inadequate No structural validity or internal consistency evaluation; psychometric evidence is minimal.
[22]	Inadequate Uses validated NOSACQ-50 without performing new expert or target-population content validation.	Inadequate The study used the NOSACQ-50 without any structural evaluation in the sample.	Inadequate α between 0.70–0.92; not validating structure.	NA	NA	NA	NA	Inadequate Consistent associations and acceptable reliability; no structural or invariance evaluation.
[36]	Inadequate Uses a validated questionnaire translated; limited content assessment beyond translation.	Inadequate No EFA/CFA/IRT; dimensionality not assessed.	Inadequate α = 0.84; structural validity not established.	NA	NA	Very good Clear responsiveness to training.	NA	Inadequate No structural, construct, or internal consistency evaluation beyond α; very limited psychometric evidence.
[24]	Inadequate Uses LSCAT and Zohar scales with face validity only; no systematic expert or population evaluation.	Inadequate Only descriptive statistics/correlations; no factor analysis or model evaluation.	Inadequate α = 0.70 (LSCAT) and 0.96 (ZSCQ); no structural confirmation.	NA	Adequate Differences between production and non-production groups;	NA	Inadequate Group comparisons only; no MG-CFA, DIF, or IRT.	Inadequate No content validity; no structural validity; analyses limited to descriptive comparisons.
[25]	Very good Validated Polish version based on structured expert review and adaptation process.	Inadequate Replicates prior EFA; no CFA/IRT performed in this sample.	Inadequate α = 0.61–0.88; some dimensions marginal, no CFA.	NA	Adequate Significant differences between occupational groups.	NA	Adequate Comparability among the three groups of workers.	Inadequate Acceptable reliability and construct associations; lacks confirmatory structural testing.
[26]	Inadequate Use of previously validated instruments with literature-based adaptation; limited direct content validation.	Very good CFA indicated a good fit; strong convergent/discriminant validity.	Very good α between 0.78 and 0.86 with a confirmed CFA model.	NA	Adequate Association with self-reported accidents.	NA	NA	Inadequate No content validity, despite strong CFA and reliability.
[27]	Very good The study followed a systematic approach for the translation, cultural adaptation, and expert review.	Inadequate EFA conducted; no CFA.	Inadequate α > 0.80; strong reliability but no CFA.	NA	Adequate Correlations with organizational variables.	NA	NA	Inadequate Acceptable structure (EFA), reliability, and construct associations; lacks deeper psychometric testing.
[28]	Inadequate Adapted from the HSE questionnaire without formal expert evaluation or cultural adaptation.	Inadequate No EFA/CFA/IRT; only correlations reported.	Inadequate α between 0.689 e 0.868; borderline reliability; no CFA.	Adequate Test–retest (n = 30; 10-day interval) with Spearman r > 0.69, indicating temporal stability; ICC not reported.	Adequate Exploratory correlations with demographic variables.	NA	NA	Inadequate No content validity assessment, no structural validity (no CFA/EFA), internal consistency not interpretable, and no construct validity testing.
[29]	Inadequate Qualitative triangulation and open-ended item generation, but no structured expert panel.	Inadequate Qualitative exploratory study; no structural model to evaluate.	Inadequate Qualitative study; no internal consistency measures.	NA	Adequate Emerging dimensions comparable to the literature.	NA	NA	Inadequate Qualitative and exploratory only; lacks quantitative psychometric evaluation.
[30]	Very good Systematic review and co-creation with HSE managers; strong evidence of content relevance and coverage.	Very good Using a sample of 1688 workers, the analysis employed both EFA and CFA, yielding eight final dimensions.	Very good α > 0.80; short version also validated.	NA	Very good Strong convergent and known-groups validity across sectors.	NA	Very good Multilevel invariance across organizational, supervisor, and peer levels.	Very good Strong structural validity, strong internal consistency, and strong construct validity; robust evidence across levels.
[31]	Inadequate No direct assessment with experts/target population; relies on prior NOSACQ-24 conceptual foundation.	Very good CFA with adequate fit (CFI ≥ 0.95; RMSEA ≤ 0.06) across multiple independent samples.	Very good α > 0.85 across all factors.	NA	Very good Correlations with accidents and risk perception.	NA	NA	Inadequate Strong structural validity; missing content validity and reliability; no content validity.
[32]	Inadequate Items adapted from validated scales; pre-test with workers, but no structured expert evaluation.	Inadequate EFA revealed three factors; no CFA.	Inadequate α between 0.78 and 0.86; although EFA only.	NA	Adequate Predictors of perceived safety performance.	Adequate Factors showed direct relationships with performance perception.	NA	Inadequate Good reliability and construct associations; lacks CFA, IRT, invariance, and test–retest analyses.
